# Increased Signal Delays and Unaltered Synaptic Input Pattern Recognition in Layer III Neocortical Pyramidal Neurons of the rTg4510 Mouse Model of Tauopathy: A Computer Simulation Study With Passive Membrane

**DOI:** 10.3389/fnins.2021.721773

**Published:** 2021-10-18

**Authors:** Attila Somogyi, Ervin Wolf

**Affiliations:** ^1^Department of Anatomy, Histology and Embryology, Faculty of Medicine, University of Debrecen, Debrecen, Hungary; ^2^Department of Emergency Medicine, University of Debrecen, Debrecen, Hungary

**Keywords:** tauopathy, mouse frontal cortex, morphology and dendritic signaling, conservation of synaptic input pattern recognition, computer simulations

## Abstract

Abnormal tau proteins are involved in pathology of many neurodegenerative disorders. Transgenic rTg4510 mice express high levels of human tau protein with P301L mutation linked to chromosome 17 that has been associated with frontotemporal dementia with parkinsonism. By 9 months of age, these mice recapitulate key features of human tauopathies, including presence of hyperphosphorylated tau and neurofibrillary tangles (NFTs) in brain tissue, atrophy and loss of neurons and synapses, and hyperexcitability of neurons, as well as cognitive deficiencies. We investigated effects of such human mutant tau protein on neuronal membrane, subthreshold dendritic signaling, and synaptic input pattern recognition/discrimination in layer III frontal transgenic (TG) pyramidal neurons of 9-month-old rTg4510 mice and compared these characteristics to those of wild-type (WT) pyramidal neurons from age-matched control mice. Passive segmental cable models of WT and TG neurons were set up in the NEURON simulator by using three-dimensionally reconstructed morphology and electrophysiological data of these cells. Our computer simulations predict leakage resistance and capacitance of neuronal membrane to be unaffected by the mutant tau protein. Computer models of TG neurons showed only modest alterations in distance dependence of somatopetal voltage and current transfers along dendrites and in rise times and half-widths of somatic Excitatory Postsynaptic Potential (EPSPs) relative to WT control. In contrast, a consistent and statistically significant slowdown was detected in the speed of simulated subthreshold dendritic signal propagation in all regions of the dendritic surface of mutant neurons. Predictors of synaptic input pattern recognition/discrimination remained unaltered in model TG neurons. This suggests that tau pathology is primarily associated with failures/loss in synaptic connections rather than with altered intraneuronal synaptic integration in neurons of affected networks.

## Introduction

Two key proteins involved in pathophysiology of Alzheimer disease (AD), the most common form of dementia, are amyloid β (Aβ) and tau proteins. These proteins are present in extracellular senile plaques and intracellular neurofibrillary tangles (NFTs), respectively (Kosik et al., 1986; Dickson et al., 1988), which are well-known pathological features of AD. Although these two culprits of AD have been identified in the 1980s (Glenner et al., 1984; Brion et al., 1986), their entire role in the etiology of this disease is still to be elucidated. Mutant tau alone was demonstrated to be involved in neurofibrillary pathology, synaptic loss, and neurodegeneration (Dickstein et al., 2010; Kopeikina et al., 2013a), but abnormal tau protein is also linked to effects of Aβ as tau protein was shown to be essential to Aβ-induced neuronal degeneration in AD (Rapoport et al., 2002; Bennett et al., 2004; Revett et al., 2013). Mutant tau, if present in addition to Aβ, increases severity of memory deficit in mice (Rhein et al., 2009). Besides AD, many other neurodegenerative disorders are also associated with tau inclusions, and these diseases are collectively called tauopathies, including frontotemporal dementia with parkinsonism linked to chromosome 17 (Hutton et al., 1998; Poorkaj et al., 1998; Spillantini et al., 1998b), Pick disease, progressive supranuclear palsy, argyrophilic grain disease, certain prion diseases, and several genetic forms of Parkinson disease (Lee et al., 2001; Ludolph et al., 2001; Tolnay and Probst, 2003; Williams, 2006). Transgenic mouse models have significantly increased our understanding on role of toxic tau protein in the development of these diseases. The rTg(tau301L 4R0N) 4510 mouse model (SantaCruz et al., 2005) expresses high levels of human tau (up to 13-fold of its normal murine level) with a mutation that has been linked to familial frontotemporal dementia, the second most prevalent neurodegenerative disease (Clark et al., 1998; Dumanchin et al., 1998; Hutton et al., 1998; Spillantini et al., 1998a). By 9 months of age, these mice recapitulate many pathological changes seen in tauopathies: tangle-like tau inclusions in their brain, neuronal and synaptic loss, atrophy of dendrites, changes in electrophysiological properties of pyramidal neurons, and signs of cognitive and motoric impairments (Ramsden et al., 2005; SantaCruz et al., 2005; Rocher et al., 2010; Crimins et al., 2012; Kopeikina et al., 2013a,b; Scott et al., 2016; Holton et al., 2020; Kubota and Kirino, 2021). *In vitro*, P301L mutation was shown directly to enhance formation of paired helical filaments and promote β-sheet structure during aggregation (Barghorn et al., 2000; von Bergen et al., 2001; Fischer et al., 2007). These pathological alterations are well documented, but the precise causal link between the tau protein and the alterations of neural activities is still not well understood.

To comprehend mutant tau-related alterations of neural activities, we need to study the changes in functional synaptic connections within such vulnerable networks, and we also need to determine the effects of mutant tau protein on the neurons, the building blocks of neural assemblies that conduct and integrate Postsynaptic Potentials (PSPs). While loss of synaptic contacts and shifting of different neurotransmitter systems in transgenic animals (Katsuse et al., 2004, 2006; Kopeikina et al., 2013a) and in humans (Callahan et al., 1999; Ginsberg et al., 2000) with tauopathies have been observed and investigated, studying possible effects of tau on individual neurons’ electrophysiological properties gained less attention. However, electrophysiological alterations in layer III neocortical pyramidal neurons have been investigated during advanced tauopathy in rTg4510 mice (Rocher et al., 2010; Crimins et al., 2012). These authors recorded electrophysiological data from individual neurons of rTg4510 and age-matched wild-type (WT) mice and found that cortical neurons in rTg4510 mice have some altered active and passive properties. On average, transgenic (TG) neurons showed depolarized resting membrane potential, increased depolarizing sag potential and increased action potential firing rate in response to current steps, all of which indicate hyperexcitability. On the other hand, these TG neurons had unaltered input resistances and membrane time constants. Following whole-cell patch-clamp recordings, all neurons were filled with biocytin and dendritic arborizations, and somata of neurons were 3-dimensionally reconstructed and subjected to morphometric analysis. Morphological analysis revealed a reduction in spine density both in apical and basal dendrites and a decrease in size of apical tuft in TG neurons, but no statistically significant difference was found in soma and total neuron surface areas, dendritic diameters, or in horizontal and vertical extents of dendrites.

These combined electrophysiological and morphological studies (Rocher et al., 2010; Crimins et al., 2012) demonstrated that in the rTg4510 mouse model, cortical TG neurons suffer from variable degree of morphological regression, and parallel with this, some electrophysiological properties of these neurons get altered in advanced tauopathy.

However, regarding intrinsic neuronal properties, it remained unclear (1) if membrane properties of WT and TG neurons differ from each other, (2) whether dendrites of WT and TG neurons differ in their dendritic signaling properties, and (3) whether WT and TG neurons are different in their synaptic input pattern recognition/discrimination abilities that are important in network functions and memory? Study of these possible tau-mediated alterations in intraneuronal properties is mandatory to understand how dysfunctions in individual neurons may contribute to pathological activities of neural networks in different forms of tauopathies.

Therefore, for the first time, we studied questions 1–3 by utilizing multicompartmental computational models of these TG and WT neurons based on spatial neuron reconstructions and electrophysiological measurements.

## Materials and Methods

### Neuron Samples

**FIGURE 1:**
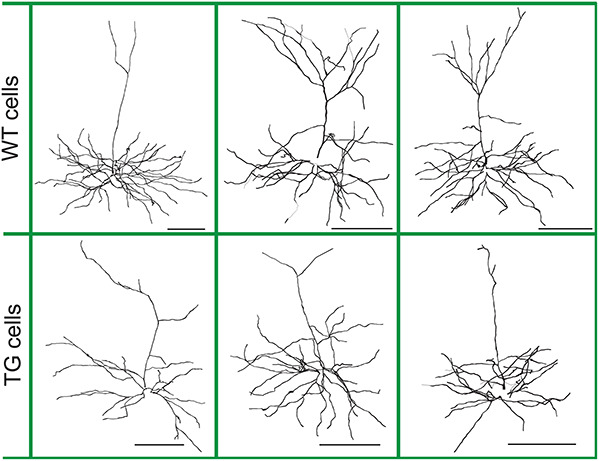
Morphology of layer III wild-type (WT) and transgenic (TG) neurons of Tg4510 mice. Representative neurons with illustrations of dendritic diameters. Neurons are from the NeuroMorpho database (neuromorpho.org) and were 3D reconstructed and analyzed morphologically and electrophysiologically by Rocher et al. (2010) and Crimins et al. (2012). Scale bars are 100 μm.

Files containing the detailed three-dimensional (3D) morphology of layer III frontal pyramidal neurons of WT and littermate age-matched rTg4510 tau mutant (P301L) TG 9-month-old mice were downloaded from the NeuroMorpho database^1^ (Rocher et al., 2010). Altogether 28 WT and 23 TG neurons were suitable for computer modeling and used for this study (Figure 1). TG neurons were analyzed whether they contain NFTs, and it was found that NFT^–^ and NFT^+^ neurons have identical somatic neuron resistance and membrane time constants and show the same general morphological characteristics (Rocher et al., 2010). Therefore, we did not distinguish NFT^–^ and NFT^+^ neurons in our study. Sex differences were not taken into account in the earlier study, where morphology and electrophysiology of these neurons were investigated; thus, we could not account for possible sex differences.

Details on tissue preparations, labeling procedures, and 3D reconstruction of neurons have been described in the earlier paper (Rocher et al., 2010). Briefly, mice were sacrificed by decapitation, and brains were placed in oxygenated (95% O_2_, 5% CO_2_) ice-cold Ringer solution. Frontal 300-μm-thick cortical slices (8–10/hemispheres) were made by a vibrating microtome and then left in oxygenated Ringer’s solution at room temperature for at least 1 h before whole-cell patch-clamp recordings. During ∼15 min long recordings slices were still superfused (2.5 ml/min) with Ringer solution. Layer III frontal (dorsal premotor) cortical pyramidal neurons were identified by infrared-differential interference contrast microscope. Passive properties were determined electrophysiologically by analyzing voltage responses to 200-ms current steps. Calculation of neuron resistance was based on slope of a regression line fitted to linear portion of the voltage–current plot. Membrane time constant was measured by fitting a single exponential function to membrane voltage in response to 10-pA hyperpolarizing current step. Following recordings, all neurons were filled with 1% biocytin; slices were fixed in 4% paraformaldehyde in 0.1 M phosphate-buffered saline for 4 days at 4°C. To allow visualization, slices were incubated in streptavidin–Alexa 546 for 2 days. Confocal images for 3D reconstructions were obtained by a Zeiss LSM-510 confocal laser-scanning microscope. Spatial reconstructions of neurons were based on integrated volumetric datasets obtained by a Volume Integration and Alignments System (VIAS) (Rodriguez et al., 2003). Z-stack stitching of 40 × confocal images was then exported to Neurolucida neuron reconstruction system with AutoNeuron and NeuroExplorer (MBF Bioscience, Williston, VT) software for automatic tracing, which were then manually corrected, and used for morphometric analysis. Spine detection and analysis were performed by the NeuronStudio on full resolution stacks by VIAS, which was followed by manual checking and corrections, if needed.

Only those neurons were included in the final dataset, which had membrane potentials ≤ −55 mV, were able to fire trains of action potentials in response to sustained depolarizing current, had well-labeled dendrites with no distal cuts, and showed intact soma.

### Compartmentalization of Neurons

**FIGURE 2:**
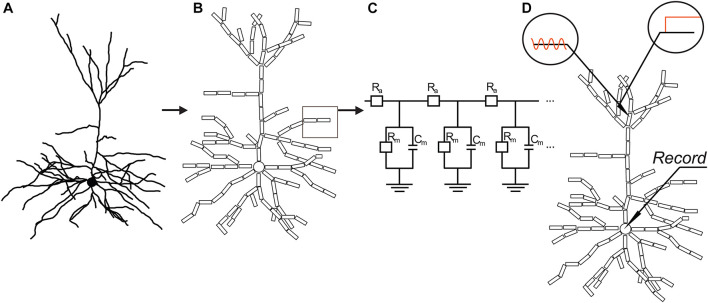
Basic concept of compartmental models used in the study. Based on 3D reconstructed morphology of neurons **(A)**, dendrites were divided into cylindrical compartments, while the soma was represented by a single spherical compartment **(B)**. Compartments were replaced and coupled together by electrical equivalent circuits **(C)** with resistors and capacitors representing specific membrane resistance (*R*_*m*_) and capacitance (*C*_*m*_), as well as axial resistance of the dendroplasm (*R*_*a*_). PSPs were simulated by injecting constant or 50-Hz sinusoid current or by conductance changes at various locations of the modeled dendrites **(D)**. Transfers, delays of simulated PSPs to the soma, and rise times/half-widths of somatic EPSPs were measured by simultaneous recordings from dendritic and somatic compartments.

Morphologically faithful compartmental models of pyramidal neurons were created in the NEURON (version 7.3–7.5) simulation environment (Hines and Carnevale, 1997, 2001) based on the neurons’ 3D data files containing length, diameter, and branching topology of dendrites (Figure 2). NEURON software numerically calculates solution of the spatially and temporally discrete approximation of linear cable equation, which has the form:


$$C_{m}\,\frac{\partial V}{\partial t}+\frac{V}{R_{m}}=\frac{r}{2\,R_{a}}\,\frac{\partial^{2}V}{\partial x^{2}}$$

where *V*, *x*, and *t* are membrane potential, location, and time; *C*_*m*_ and *R*_*m*_ are specific membrane capacitance and resistance, respectively, *R*_*a*_ is the axial resistivity, and *r* is radius.

Model neurons had single soma compartments with individually assigned soma surface areas according to reconstructions of respective cells. These mean soma surface areas were 146.2 ± 26.3 and 205.2 ± 41.4 μm^2^ for WT and TG neurons, respectively. The numbers of dendritic compartments in model neurons were between 17 and 79 (25–66) in WT and 15 and 81 (25–60) in TG neurons in their apical and (basal) dendritic arbors, depending on the complexity of arborization pattern and size of dendrites of individual neurons.

### Modeling Spines

As loss of dendritic spines is one element of dendritic atrophy in the rTg4510 mouse model (Rocher et al., 2010; Crimins et al., 2012) and also in human tauopathies (Ferrer and Gullotta, 1990; Ferrer et al., 1990), we considered the effects of spines on dendritic impulse propagation in our computational models. Linear spine densities of model neurons were 1.25 and 1.00 spines/μm for the WT and TG neurons, respectively (Crimins et al., 2012) with common 1.5-μm^2^ spine surface areas (Larkman et al., 1992; Middei et al., 2008). Spines were not modeled as individual structures but by proportionally increasing specific membrane capacitance and conductance of dendritic compartments. This way, we accounted for electrical effects caused by increase in dendritic surface area due to dendritic spines without actually modifying length and diameter of cylindrical compartments in our computational models (Shelton, 1985; Holmes, 1989; Larkman, 1991; Jaslove, 1992; Stuart and Spruston, 1998; Schmidt-Hieber et al., 2007; Somogyi et al., 2016). In the first step of this procedure, relative increase in total surface area (*q*) caused by spines was calculated for each dendritic compartment according to the following formula: *q* = (*A*_*s*_ + *A*_*d*_)/*A*_*d*_, where *A*_*s*_ is total surface area of spines received by the dendritic compartment, and *A*_*d*_ is surface area of the “smooth” cylindrical compartment without spines. Then, the adjusted specific membrane capacitance (*C*_*m*_^∗^) and conductance (G_*m*_^∗^) of the compartment were assigned individually for each compartment as *C*_*m*_^∗^ = *C*_*m*_. q and *G*_*m*_^∗^ = *G*_*m*_. *q*, where *C*_*m*_ and *G*_*m*_ are measurable capacitance and conductance for unit membrane area of the spiny neuron.

Regarding the many thousands of spines per neuron, such modeling is feasible as we were not interested in signal propagation within spines but wanted to explore possible changes in dendritic impulse propagation along dendrites by taking effects of spine loss into consideration.

### Membrane Models of Neurons

Details of distribution and kinetics of various ion channels over somadendritic surface area of layer III pyramidal neurons of WT and Tg4510 mice are not available. Therefore, we restricted our investigations to passive membrane, and in our canonical membrane model, specific resistance and capacitance of the plasma membrane (transmembrane resistance and capacitance for the unit membrane area) were considered to be uniformly distributed over soma and dendritic compartments. To set specific membrane resistances (*R*_*m*_) of neurons in the computer model, first specific resistance was varied by hand in each WT and TG neuron model until the electrophysiologically determined mean somatic neuron resistance (197 ± 23 MΩ for WT and 228 ± 23 MΩ for TG cells) was matched (Rocher et al., 2010). During these simulations, somatic neuron resistance (*R*_*in*_) was computed by measuring voltage changes at the soma in response to constant current injection (*R*_*in*_ = ΔV/I, where Δ*V* is amplitude of somatic depolarization, and *I* is injected current), just like in real electrophysiological experiments.

In the second step of our manual fitting procedure, specific membrane capacitance (*C*_*m*_) was set by varying *C*_*m*_ (with the *R*_*m*_ found in the previous step of fitting) until simulated membrane time constants (*τ*) matched those measured electrophysiologically in WT (32.5 ± 4.1 ms) and rTg4510 (35.2 ± 3.4 ms) mice (Rocher et al., 2010). Simulated membrane time constants were calculated from voltage responses of model neurons to depolarizing current steps by measuring time needed for the soma of model neurons to reach 63% of peak depolarization. Axial resistance (*R*_*a*_) of cytosol was 150 Ωcm (Trevelyan and Jack, 2002; Kabaso et al., 2009) in all model neurons. Integration time was 0.025 ms in all simulations (Hines and Carnevale, 1997, 2001). A summary of model parameters is presented in Table 1.

**TABLE 1:** Summary of fixed morphological and physiological data used as input parameters of the model and free parameters computed by fitting procedures, as well as a set of simulation output data.

Fixed parameters			Free parameters	Simulation output
Name	Value	Source		
3D dendritic trajectory and soma	From individual data files	Neuromor pho.org	Specific membrane capacitance (μF/cm^2^)	Transfers
Spine density (1/μm)	1.25 (WT); 1.0 (TG)	Crimins et al., 2012	Specific membrane resistance (Ω⋅cm^2^)	Rise times
Spine surface area (μm^2^)	1.5	Larkman et al., 1992; Middei et al., 2008		Half-widths
Time constant (ms)	32.5 ± 4.1 (WT); 35.2 ± 3.4 (TG)	Rocher et al., 2010		Delays
Input resistance (MΩ)	197 ± 23 (WT); 228 ± 23 (TG)	Rocher et al., 2010		Electrotonic distances
Axial resistance (Ω⋅cm)	150	Trevelyan and Jack, 2002; Kabaso et al., 2009		
Resting membrane potential (mV)	−75 (WT); −65 (TG)	Rocher et al., 2010		
Alpha synapse max conductance (nS)	0.25	Sarid et al., 2007		
Peak time of alpha synapse conductance (ms)	0.5	Sarid et al., 2007		
Integration time step (ms)	0.025	Hines and Carnevale, 1997, 2001		

### Initiation of Postsynaptic Potentials and Measures of Dendritic Signal Transfer

Postsynaptic potentials were simulated by steady-state or sinusoidal (50 Hz) current injections to dendritic sites or by local dendritic conductance changes according to an alpha function. The alpha function has the following form:


g(t)syn=g⋅maxt/t⋅peakexp(1-t/t)peak

where g_syn_ and g_max_ are actual and maximum synaptic conductance and t_peak_ is the time when the conductance has its maximum value (g_max_), t is time. In our simulations to mimic AMPAR-mediated single-synapse conductance changes g_max_ was 0.25 nS and t_peak_ was 0.5 ms (Sarid et al., 2007). Thus, value of synaptic current (I_syn_) is


I(V,t)syn=g(t)syn⋅(V(t)-E)syn

where *E*_syn_ and *V* are reversal potential, taken as 0 mV, and the membrane potential, respectively. Resting membrane potentials in simulations were −75 mV for WT, and −65 mV for TG neurons (Table 1; Rocher et al., 2010).

Distance between neighboring injection sites (modeled synaptic loci) was never farther than 37 μm or 0.2 space constant resulting in 83–270 injection sites per neuron, depending on size and arborization pattern of dendrites.

Subthreshold somatopetal dendritic impulse propagation was studied and compared in models of TG and WT neurons by analyzing current transfers, steady-state and sinusoidal voltage transfers, and delays of dendritic PSPs, generated by local current injections. In addition, 10–90% rise times and half-widths of somatic Postsynaptic Potentials (EPSPs) were also studied, whereas dendritic PSPs were simulated by local dendritic conductance changes. *Voltage transfer* was defined as the ratio of amplitudes of somatic and dendritic PSPs measured during dendritic current injections. *Current transfer* was defined as the fraction of electrical charge that reached the soma relative to the total charge injected at the dendritic site. *Total delays* associated with propagation of PSPs between dendritic points and the soma were measured as sum of time needed for injected current to develop a local dendritic PSP (local delay) and the time needed for this locally developing potential change to reach the soma (propagation delay). *Local delay* was quantified as the time delay between centroids of voltage–time and current–time curves at the site of current injection. *Propagation delay* was computed as the time difference between centroids of the voltage–time curves at the soma and at the dendritic injection site (Agmon-Snir and Segev, 1993; Zador et al., 1995). This way, total delay (delay from this point on), which is the sum of local and propagation delays, measures total time that elapses between synaptic current flow at a working dendritic synapse and the development of somatic voltage response.

In order to compare dendritic impulse propagation in WT and TG neurons, two types of comparison graphs were created. (1) To assess transfers/delays of propagating PSPs and rise times/half-widths of somatic EPSPs in the function of site of PSP generation in WT and TG neurons, these descriptors of dendritic impulse propagation were graphed as a function of path distance of the site of PSP generation (as a function of synaptic loci) measured from the soma. (2) In addition, to assess relative weight of any given descriptor among the many location-dependent values, distributions of dendritic surface areas (a good approximation of distributions of synapses received by dendrites; see later) were graphed in the function of these descriptors of dendritic impulse propagation. To account for the different degrees of morphological alterations seen in apical and basal arbors of TG neurons (Rocher et al., 2010; Crimins et al., 2012), the two types of comparison graphs were computed for apical and basal dendritic arbors separately in both WT and TG neurons.

To quantify the size overall alteration in each descriptor of dendritic impulse propagation in TG neurons, area weighed arithmetic means of the descriptors were also computed for each WT and TG neuron. The formula used in these calculations is Σ*A*_*i*_ ⋅ *D*_*i*_ / Σ*A*_*i*_, where *A*_*i*_ is the surface area of the *i*th dendritic segment and *D*_*i*_ is the value of descriptor describing somatopetal propagation of the PSP when the PSP was initiated at midpoint of the *i*th segment, and Σ*A*_*i*_ is the total surface area of dendrites in the subject neuron.

Finally, to test whether our major findings are independent of the natural within-group variations, both types of comparison graphs were created and analyzed on normalized scales as well. In these normalized graphs, distance dependence and weight of descriptors were analyzed over a normalized path distance scale and over normalized scale of descriptors, respectively. Normalized path distances and descriptors were measured as percentage of maximum path distances and percentage of maximum values of descriptors that occur in different neurons.

### Measures of Synaptic Input Pattern Recognition Capabilities in Wild-Type and Transgenic Neurons

Synaptic input pattern recognition/differentiation capabilities in WT and TG neurons were compared by predictors of these capabilities based on a detailed study (de Sousa et al., 2015) involving hundreds of thousands of model neurons with different morphologies and with passive and active membrane conductances. These authors used a Hebbian learning rule (use-dependent synaptic facilitation) in their computational model. Different, randomly chosen sets of synapses were activated over dendrites of a postsynaptic neuron, and synapses that were activated multiple times during this “training phase” became stronger (their synaptic conductance was gradually increased). Following this “training phase,” a new “novel” pattern of synapses was activated, and the difference between recognition of “learnt” and “novel” patterns (synaptic input pattern discrimination) was quantified. This quantification was based on the ratio of somatic EPSPs or, in case of active membranes, by the ratio of the number of spikes produced by the postsynaptic neuron in response to activation of the respective synaptic patterns. It was found (de Sousa et al., 2015) that synaptic input pattern recognition/discrimination was inversely proportional to two simple metrics, mean electrotonic distance of synapses, and within-cell variance of these electrotonic distances, in neurons with both passive and active dendritic membranes.

Based on this thorough analysis, we computed these two metrics in each WT and TG neuron. Then, we used these values as predictors to test if synaptic input pattern recognition/discrimination gets altered in TG neurons affected by mutant tau. Mean and variance of electrotonic distances of synapses were estimated from electrotonic distances of dendritic injections sites (loci of simulated synapses) from the soma in each model neuron.

To estimate mean electrotonic distance of synapses, electrotonic distances of midpoints of dendritic segments from the soma were averaged in each WT and TG neuron individually according to the following formula: 1/*n* Σ *L*_*i*_, where *n* is number of dendritic segments in the neuron, and *L*_*i*_ is the electrotonic distance of the midpoint of the *i*th dendritic segments from the soma. *L*_*i*_ = Σ (*l*_*k*_ / λ_*k*_), where *l*_*k*_ is geometrical length of the *k*th segment on route between the soma and the midpoint of the *i*th segment, and λ_*k*_ is the space constant of the *k*th segment. The space constant of the *k*th segment is λ_*k*_ = (*d*_*k*_ ⋅ *R*_*mk*_/4 ⋅ *R*_*a*_)½, where *d*_*k*_ and *R*_*mk*_ are the diameter and specific dendritic membrane resistance of the *k*th segment after correction for the spine surface area of that segment, and *R*_*a*_ is axial resistance.

### Statistical Analysis

Statistical tests were performed, and figures were plotted by using the Microsoft Office (Microsoft Corp.), PAST (Hammer et al., 2001), and SigmaPlot for Windows version 14 (Systat Software, Inc., San Jose, CA, United States) software. Identity of distributions of dendritic lengths and dendritic surface areas was tested by a Kolmogorov–Smirnov test. For pairwise comparisons of electrotonic distances and their variances, specific membrane resistances, and capacitances of WT and TG neurons, Mann–Whitney test was used. Comparison graphs showing distance dependence of descriptors of propagating PSPs and dendritic surface areas with identical descriptors were compared by two-way analysis of variance (ANOVA) tests to reveal overall alterations in dendritic signaling in TG neurons. Bonferroni *post hoc* tests were used to identify path distance regions or intervals of descriptors where WT and TG neurons differ. These *post hoc* tests were performed only in intervals with at least three data from both populations of neurons. Level of statistical significance was 0.05 in all cases. Percentage of distance regions and descriptor intervals with statistically significant differences between WT and TG neurons was used to quantify the degree of alterations in dendritic signaling.

## Results

### Distribution of Dendritic Surface Area as a Function of Path Distance From the Soma Approximates Distribution of Excitatory Synapses Received by Dendrites of Wild-Type and Transgenic Pyramidal Neurons

Based on observations that mutant human tau protein alters morphology of pyramidal neurons, we aimed to explore some of the possible diverse functional consequences of tau burden on pyramidal neurons. More specifically, we wanted to characterize features of subthreshold alterations of intradendritic signaling in pyramidal neurons of rTg4510 mice. Quantification and comparison of dendritic impulse propagation were achieved by using comparison graphs. In these graphs, different descriptors (transfers, delays of propagating dendritic PSPs and rise times, and half-widths of somatic EPSPs) of somatopetal dendritic signaling were depicted in function of path distance of the simulated synaptic site from the soma. To account for the many different values of location-dependent descriptors, we also wanted to give weights to them, representing the fraction of dendritic synapses with similar descriptors. One way to estimate fraction of synapses with similar descriptors is to use the fraction of dendritic surface area from where PSPs have similar descriptor values as they travel toward the soma. To justify the use of this fraction of dendritic surface area for the approximation of dendritic synapses with similar descriptors, we addressed the question of how good this approximation can be.

**FIGURE 3:**
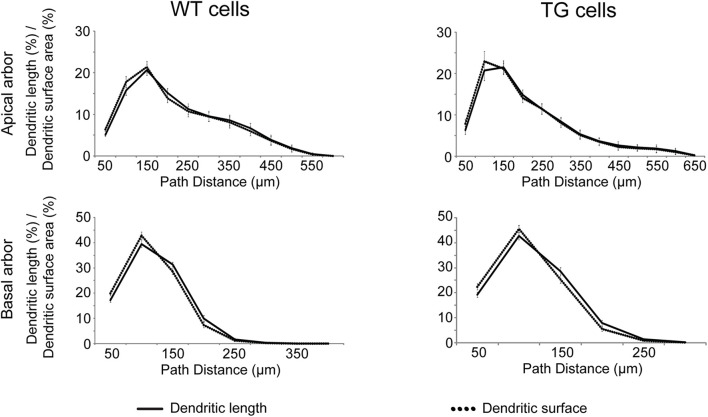
Distributions of dendritic length (solid lines) and surface area (dotted lines) as a function of path distance measured from the soma. Distributions are statistically identical (Kolmogorov–Smirnov test, *p* > 0.999 both in apical (upper panels) and basal (lower panels) dendritic arbors of WT (left panels) and TG (right panels) neurons.

In neocortical pyramidal neurons, more than 90% of excitatory synapses is received by dendritic spines, and in the most cases, a single spine receives one synapse only (Nimchinsky et al., 2002). It was also shown that only 3.6% of spines do not receive synapses in mouse neocortex (Arellano et al., 2007). These observations suggest that distribution of spines could be a good estimate for distribution of excitatory synapses over the dendrites. As linear spine density (number of spines along the unit length of dendrite) is nearly constant in layer III pyramidal neurons of WT and rTg4510 mice (Rocher et al., 2010; Crimins et al., 2012), distribution of dendritic length provides a good estimate of distribution of spines and excitatory synapses as well. We compared the fraction of dendritic length and dendritic surface area as a function of path distance from the soma, and these distributions were statistically identical in WT and TG neurons (Figure 3, Kolmogorov–Smirnov test, *p* > 0.999). This means that the percentage of total dendritic surface area within a path distance range could be used for estimating percentage of total number of excitatory synapses received by the respective dendritic surface.

Distribution of inhibitory synapses cannot be related directly to distribution of spines as they are usually received by dendritic shaft. However, the ratio of symmetrical to asymmetrical synapses was found to be nearly constant on dendritic shafts of different regions of reconstructed neocortical dendrites in mouse, and the ratio of the total number of symmetrical and asymmetrical synapses was also constant (Hersch and White, 1982). Many other studies have also shown evidence for close interrelationship between the size of dendritic receptive surface, number of synapses, and number of spines (Colonnier, 1968; Feldman, 1975; Feldman and Dowd, 1975; Muller et al., 1984).

Based on these arguments, percentages of total dendritic surface area of neurons were used to estimate fractions of total number of synapses generating PSPs with similar descriptors.

### Passive Membrane Properties Remain Unaltered in Transgenic Neurons

**FIGURE 4:**
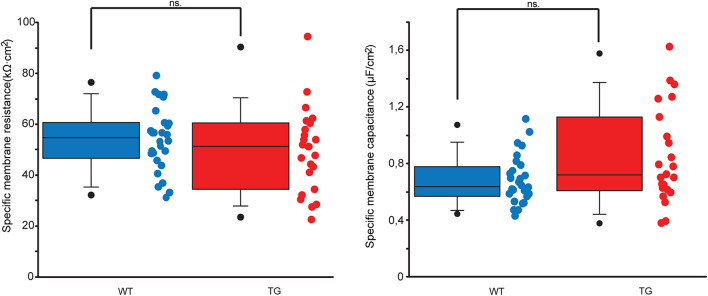
Estimated specific membrane resistances and capacitances of WT and TG neurons. Dot and box plots comparing specific membrane resistances (left panel) and capacitances (right panel) of WT (blue) and TG (red) neurons. None of these membrane parameters get altered significantly by the mutant tau protein (Mann–Whitney test, *p* = 0.267 and *p* = 0.083 for resistances and capacitances, respectively). Lower and upper boundaries of the boxes indicate the 25th and 75th percentiles; a line within each box marks the median value; whiskers below and above the boxes show the 10th and 90th percentiles; lower and upper closed circles represent the 5th and 95th percentiles.

To set up segmental cable models of WT and TG neurons, we had to estimate resistance and capacitance of a unit area of neuronal membrane, which are currently not directly available from electrophysiological experiments. These estimates were carried out in anatomically faithful compartmental models of the neurons and were based on fitting somatic DC neuron resistances and membrane time constants measured electrophysiologically (see section “Materials and Methods”). Estimated specific resistances and capacitances in TG neurons were proven to be identical to those in WT neurons (Mann–Whitney test, *p* = 0.267 and *p* = 0.083), which were not affected by the mutant tau protein and used as control (Figure 4).

### Rationale of Analysis of Dendritic Signaling

Significantly diverse tau-induced morphological alterations combined with the unaltered passive membrane properties in TG neurons led us to analyze possible functional consequences of observed morphological alterations on intraneuronal dendritic signaling. In this analysis, we utilized two types of comparison graphs: one type shows location dependence of propagation-related descriptors of locally generated dendritic PSPs, and the other type shows proportion of dendritic synapses (estimated as the proportion of total dendritic surface area) with similar values of descriptors of dendritic signaling. This latter type of comparison graph was meant to estimate the weight of any given value of a signaling property among the many different location-dependent values. Rationale of this is that if either the location dependence of signal transfers along the dendrites or proportion of synapses with bigger or smaller transfers (bigger or smaller ability to affect the soma potential) gets significantly altered in TG neurons, then this alteration is considered as tau-induced modification in dendritic signaling relative to control, WT neurons. Idea behind a similar analysis of delays and rise times and half-widths of somatic EPSPs is the same. Timing and shape of somatic EPSPs can also affect action potential generation. Thus, if either location dependence of these descriptors or fraction of synapses with bigger or smaller descriptors changes significantly in TG neurons, then such a change is treated as an indication of an altered intrinsic signaling mechanism in the affected neuron in response to toxic effect of mutant tau protein. Both types of comparison graphs were created for apical and basal dendritic arbors separately and were graphed over normalized and absolute scales as well.

### Significance of Different Measures of Dendritic Impulse Propagation

*Steady-state voltage transfer* measures the extent to which a dendritic synapse with relatively slow time course [e.g., those mediated by *N*-methyl-D-aspartate (NMDA) receptors] can influence membrane potential dynamics at the soma (at the nearby axon hillock). Synapses at loci with higher steady-state voltage transfers may have bigger influence in shaping somatic potential and therefore firing activity of the postsynaptic neuron than synapses at loci with smaller transfer values (assuming other factors are equal).

Voltage transfers depend on the frequency of signal because of non-zero membrane capacitance. As a consequence of this, PSPs with short time constants (e.g., those mediated by AMPA receptors) attenuate differently than PSPs with slower kinetics. Therefore, we extended our steady-state voltage transfer analysis with computation of the 50-Hz sinusoid voltage transfer profiles.

In some cases, predominantly when voltage perturbations are small, the amount of electrical charge reaching the soma predicts chances for firing an action potential better than amplitude of somatic membrane potential (Jack et al., 1975). To account for this notion, current transfer was measured as fraction of electric charge reaching the soma relative to the total charge injected locally at the locus of modeled dendritic synapse. Other interpretation of this measure is based on equality between the somatopetal charge transfer from a dendritic point to the soma and somatofugal voltage transfer from the soma to the same dendritic point (Zador et al., 1995). This equality is held in any dendritic tree and is valid for any dendritic point. Therefore, somatopetal current transfer may be interpreted as a measure of efficiency of passive back-propagation of action potentials along the dendrites.

Possible tau-induced alterations in delays of PSPs or in shape of somatic PSPs can also affect summation of dendritic signals arriving at the cell body. Summation of PSPs arriving at the soma in response to a given pattern of simultaneous synaptic inputs to a neuron may be different if delays of PSPs or the shape of summating somatic PSPs gets altered. In case delays of PSPs of certain local synapses get too long relative to delays of PSPs generated by other synapses, then these modifications may prevent delayed PSPs from summating effectively with other simultaneously generated PSPs that arrive at the soma much earlier. If half-width of somatic PSPs gets increased, time window for effective summation of multiple PSPs arriving at the soma will widen, and coincidence detection capability will worsen. Finally, longer rise times may postpone action potential generation by delaying somatic membrane potential to reach the voltage threshold.

### Alterations in Dendritic Signaling of Transgenic Neurons

#### Transfers of Postsynaptic Potentials

**FIGURE 5:**
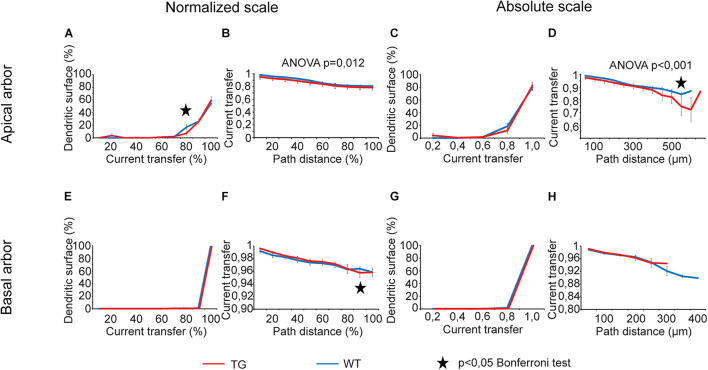
Alterations in current transfers of locally generated PSPs in advanced tauopathy. Comparison graphs showing alterations in dendritic impulse propagation of TG (red lines) neurons relative to control, WT neurons (blue lines). Two types of comparison graphs are presented: one of them shows percentages of dendritic surface area (an approximation of the fraction of dendritic synapses) with similar transfers of locally generated PSPs **(A,C,E,G)**; the other one displays location dependence of the current transfer **(B,D,F,H)**. Comparison graphs were computed both along absolute (right half) and normalized scales (left half), where path distances and transfers were measured as percentages of their maximum values. Apical (upper panels) and basal (lower panels) dendritic arbors were analyzed separately. Overall statistical evaluations of comparison graphs were made by two-way ANOVA tests. Significance levels of these ANOVA tests were indicated where statistically significant overall alteration in comparison graphs was found (elsewhere *p* > 0.05). Asterisks mark path distance regions and transfer intervals with statistically significant differences between WT and TG neurons (Bonferroni *post hoc* test, *p* < 0.05). Distance regions and transfer intervals with less than 3–3 data points in TG-WT comparisons were omitted from statistical tests.

Dendritic signaling was studied by analysis of eight comparison graphs for each transfer property (current transfer and steady-state and sinusoid voltage transfers) and for delays of PSPs. In case of current transfer, two of eight comparison graphs showed statistically significant overall tau-induced alteration (Figure 5, two-way ANOVA, *p* = 0.012 and *p* < 0.001, location dependence in apical dendrites over normalized and absolute scales, respectively).

**FIGURE 6:**
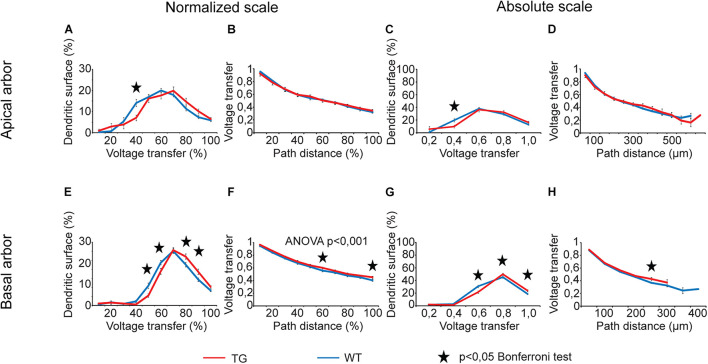
Alterations in steady-state voltage transfers in advanced tauopathy. Comparison graphs showing alterations in steady-state voltage transfers of TG (red lines) neurons relative to control, WT neurons (blue lines). Overall statistical evaluations of comparison graphs were made by two-way ANOVA tests. Significance levels of these ANOVA tests were indicated where statistically significant overall alteration in comparison graphs was found (elsewhere *p* > 0.05). Asterisks mark path distance regions and transfer intervals with statistically significant differences between WT and TG neurons (Bonferroni *post hoc* test, *p* < 0.05). Distance regions and transfer intervals with less than 3–3 data points in TG-WT comparisons were omitted from statistical tests.

**FIGURE 7:**
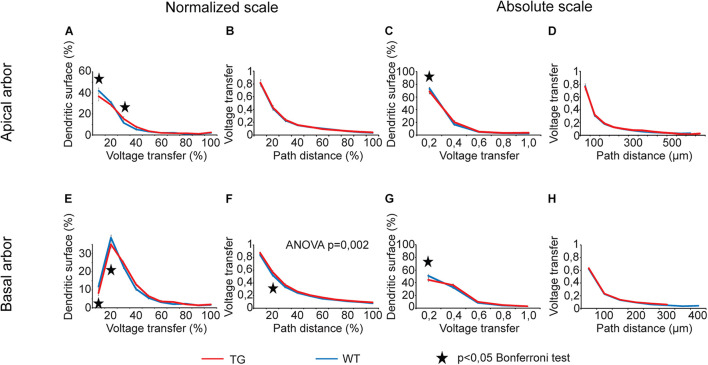
Alterations in sinusoid voltage transfers in advanced tauopathy. Comparison graphs showing alterations in sinusoid voltage transfers of TG (red lines) neurons relative to control, WT neurons (blue lines). Overall statistical evaluations of comparison graphs were made by two-way ANOVA tests. Significance levels of these ANOVA tests were indicated where statistically significant overall alteration in comparison graphs was found (elsewhere *p* > 0.05). Asterisks mark path distance regions and transfer intervals with statistically significant differences between WT and TG neurons (Bonferroni *post hoc* test, *p* < 0.05). Distance regions and transfer intervals with less than 3–3 data points in TG-WT comparisons were omitted from statistical tests.

For voltage transfers, there was only one comparison graph with statistically significant overall alteration for steady-state signals (Figure 6) and one for sinusoidal signals (Figure 7, two-way ANOVA, *p* < 0.001 and *p* = 0.002 for location dependence of steady-state and sinusoid voltage transfers in basal dendrites). All these comparison graphs on current and voltage transfers with statistically significant overall differences between WT and TG neurons showed alterations in distance dependence of transfers, and no comparison graph showed statistically significant overall alteration in the distribution of dendritic surface area (distribution of synapses) as a function of current or voltage transfer neither in apical nor in basal dendritic arbors (two-way ANOVA, *p* > 0.05).

During analysis of comparison graphs with distance dependence of voltage and current transfers, Bonferroni *post hoc* tests detected only one or two distance regions per graph with significantly altered transfers in TG neurons, indicating spatially restricted alterations in transfers. At distances where transfers in WT and TG neurons were found to be significantly different, the steady-state and sinusoid voltage transfers were always bigger, and current transfers were always smaller in TG neurons relative to control, regardless that absolute or normalized path distance scales were considered.

For distributions of dendritic surface area as a function of transfers, ANOVA tests did not show any statistically significant overall alteration in TG neurons (*p* > 0.05). However, Bonferroni *post hoc* tests revealed a reorganization of dendritic surface. In TG neurons, smaller fraction of dendritic surface area (smaller fraction of synapses) is associated with lower transfers, and bigger fraction is associated with higher transfers. Such a reorganization means that the average transfer value of multiple PSPs generated by localized synaptic activities may get bigger under the effect of mutant human tau protein, potentially increasing the general excitability of TG neurons. This modification in distribution of dendritic surface area (distribution of synapses) was consistently present both in apical and basal dendritic arbors and over normalized and absolute scales as well. Alteration was the most obvious in case of steady-state voltage transfers of basal dendritic arbors (Figures 6E,G), where such a rearrangement in distribution of dendritic receptive field led to statistically significantly bigger area weighted average steady-state voltage transfer in TG neurons (0.68 ± 0.01 vs. 0.65 ± 0.01 in TG vs. WT neurons, *p* = 0.016, Mann–Whitney test). This better voltage transfer is in line with increased general excitability of neurons seen in mutant tau-TG animals (Rocher et al., 2010; Crimins et al., 2012) and during seizures in humans (Sanchez et al., 2018) with tauopathies.

#### Delays of Postsynaptic Potentials

**FIGURE 8:**
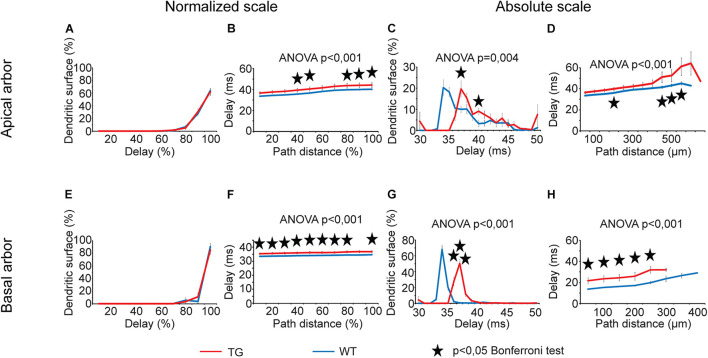
Alterations in delays of PSPs in advanced tauopathy. See legend for analog Figure 5 with the difference that instead of transfers; here time delays of individually generated PSPs were graphed. For better visibility, distribution of dendritic surface area over absolute scale of delays is shown for the 30–50-ms interval only, where statistically significant alterations in TG neurons were detected. Outside this delay range, fractions of dendritic surface area quickly converge to zero in both TG and WT neurons. Overall statistical evaluations of comparison graphs were made by two-way ANOVA tests. Significance levels of these ANOVA tests were indicated where statistically significant overall alteration in comparison graphs was found (elsewhere *p* > 0.05). Asterisks mark path distance regions and transfer intervals with statistically significant differences between WT and TG neurons (Bonferroni *post hoc* test, *p* < 0.05). Distance regions and transfer intervals with less than 3–3 data points in TG-WT comparisons were omitted from statistical tests. TG and WT neurons are red and blue lines, respectively.

Both distance dependence of delays and distributions of dendritic surface areas showed statistically significant overall alterations in TG neurons (Figure 8, two-way ANOVA, *p* < 0.001 and *p* = 0.004) except for the distribution of dendritic surface area along normalized scale. We detected a general and statistically significant slowdown in dendritic signaling (increase in delays) both in apical and in basal dendritic arbors of TG neurons along normalized and absolute path distance scales too (two-way ANOVA, *p* < 0.001). A rearrangement of the distribution of dendritic surface area according to delays was also revealed along absolute scale of delays and this alteration was present in the apical and basal arbors as well (two-way ANOVA, *p* = 0.004 and *p* < 0.001). Such a rearrangement increased the fraction of dendritic surface area in TG neurons where locally generated PSPs are associated with longer delays during their propagation toward the cell body (Bonferroni test, *p* < 0.05). In other words, alteration in distribution of dendritic surface area in TG neurons showed a shift toward bigger delays, indicating presence of a bigger percentage of synapses at loci associated with longer delays of PSPs (Figures 8C,G). This led to a statistically significant increase in area weighted average delays of PSPs both in apical and basal dendrites of TG neurons relative to control (40.6 ± 1.90 ms vs. 37.1 ± 0.45 ms in apical and 35.9 ± 0.75 ms vs. 33.8 ± 0.13 ms in basal dendrites, Mann–Whitney test, *p* = 0.003 and *p* < 0.001).

#### Rise Times and Half-Widths of Somatic Excitatory Postsynaptic Potential

**FIGURE 9:**
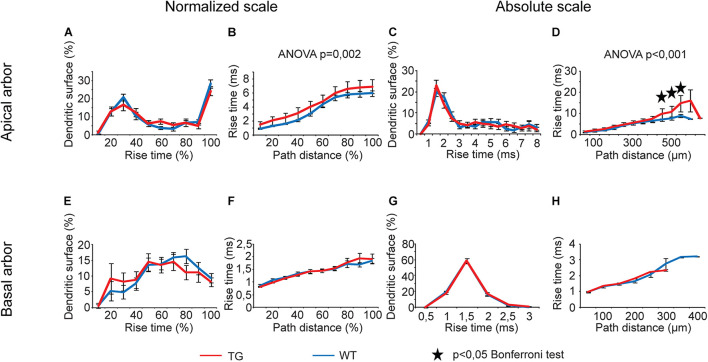
Alterations in rise times of somatic EPSPs in advanced tauopathy. Dendritic PSPs were generated by local dendritic synaptic conductance changes at different distances from the soma, according to an alpha function with kinetic parameters corresponding to AMPAR-mediated single-synapse activities. For better visibility, distribution of dendritic surface area over absolute scale of rise times is shown up to rise times where at least 3–3 data points for WT (blue lines) and TG neurons (red lines) were present and statistical tests could be performed. Outside this range, percentages of dendritic surface areas converged to zero. Asterisks mark path distance regions where simulated PSPs, after traveling to the soma, led to statistically significant rise times of somatic EPSPs in WT and TG neurons (Bonferroni *post hoc* test, *p* < 0.05).

Rise times of simulated somatic EPSPs as a function of path distance showed statistically significant overall alterations when PSPs started from apical but not from basal dendrites of TG neurons. This was the case both on relative and absolute scales of distances (Figures 9B,D, two-way ANOVA, *p* = 0.002 and *p* < 0.001). Rise times of somatic EPSPs were invariably longer in TG neurons regardless the distance region where dendritic PSP was initiated, but these changes reached statistically significant levels (Bonferroni test, *p* < 0.024) in the 450- to 550-μm distance region only (Figure 9D). Distributions of dendritic surface area as a function of rise time did not get altered in TG neurons (two-way ANOVA, *p* > 0.05).

**FIGURE 10:**
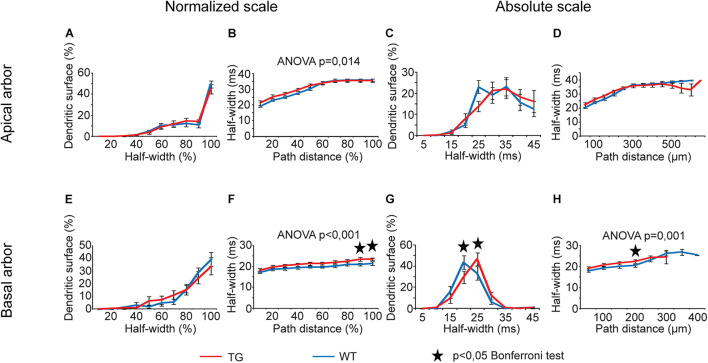
Alterations in half-widths of somatic EPSPs in advanced tauopathy. Half-widths of somatic EPSPs, in response to locally generated dendritic synaptic conductance changes at different distances from the soma, were compared in WT and TG neurons (blue and red lines, respectively). These conductance changes mimicked AMPAR-mediated single synaptic activities. Asterisks mark path distance and half-width intervals where simulated PSPs, after traveling to the soma, led to statistically significant half-widths of somatic EPSPs in WT and TG neurons (Bonferroni *post hoc* test, *p* < 0.05).

Analysis of half-widths of somatic EPSPs showed statistically significant overall alterations only in the function of path distances of the site of PSP initiation (Figures 10B,F,H, two-way ANOVA, *p* = 0.014, *p* < 0.001, and *p* = 0.001, respectively), but distributions of dendritic surface areas as a function of half-widths exhibited no overall alteration (two-way ANOVA, *p* > 0.05). Wherever Bonferroni *post hoc* test detected significant alteration in lengths of half-widths of somatic EPSPs starting from a given distance region, this alteration was always a lengthening of the half-width when PSPs started from the basal dendritic arbors of TG neurons (Figures 10F,H). In addition, *post hoc* tests revealed a shift in distribution of dendritic surface areas toward longer half-widths in basal arbors of mutant tau-affected neurons (Bonferroni test, *p* = 0.035 and *p* = 0.046; Figure 10G).

Area weighed mean rise times and half-widths of somatic EPSPs increased for PSPs arriving from apical and basal arbors of TG neurons relative to WT control, but these increases never represented statistically significant difference between WT and TG neurons (Mann–Whitney test, *p* > 0.05).

### Differential Mutant Human Tau-Induced Alterations in Dendritic Signaling of Neurons

**FIGURE 11:**
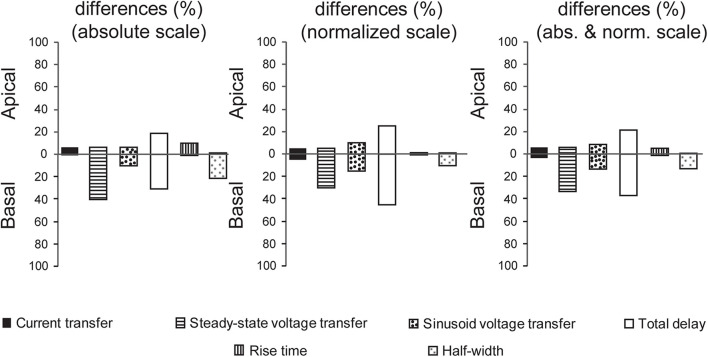
Comparisons of mutant human tau-induced alterations in dendritic signaling. Percentage of intervals where significant alterations in dendritic signaling were detected in TG neurons along absolute and normalized scales of comparison graphs as well as grand summary combining alterations detected along both scales. Upper and lower extensions of bars show alterations in apical and basal dendritic arbors, respectively. Data shown here are based on comparison graphs presented in Figures 5–10. The total number of intervals with at least 3–3 data points for WT and TG neurons was taken as 100%.

Vulnerabilities of layer III pyramidal neurons to different, human mutant tau-induced alterations of dendritic impulse propagation were quantified and compared. This analysis was based on comparison graphs (Figures 5–10), where intervals with statistically significant differences (Bonferroni test, *p* < 0.05) between descriptors of dendritic signaling of WT and TG neurons were marked by asterisks. We counted the number of intervals, where statistically significant difference was detected between WT and TG cells in location dependence of a given descriptor plus in distribution of dendritic surface area as a function of that descriptor. These counts were then converted to percentages taking the total number of intervals where statistical tests could be performed (there were at least 3–3 data points for WT and TG neurons) as 100%. These calculations were performed separately for comparison graphs with absolute and normalized scales and by combining results along both scales (Figure 11).

A general observation shown by Figure 11 is different vulnerability of apical and basal dendritic arbors to tau-induced alterations in dendritic signaling. The biggest contrast in vulnerabilities of apical and basal arbors was detected when mutant-tau–induced alterations of half-widths of somatic EPSPs were investigated. In this case, half-widths were significantly altered in TG neurons relative to WT control only if PSPs started from the basal arbor, whereas no alteration was found when PSPs started from any region of apical arbors. Generally, basal arbors suffer from a higher degree of alterations in other descriptors of dendritic impulse propagation too (the only exception is current transfer and rise time of somatic EPSPs, whose alterations were the smallest among the descriptors). This is in line with morphological observation that basal dendritic arbors of pyramidal neurons get altered earlier during the course of tau-induced neurotoxicity than the apical arbors in rTg4510 mice (Crimins et al., 2012) suggesting different degrees of vulnerabilities of the two dendritic arbors.

Regarding *variable degrees of alterations in different descriptors* of dendritic signaling, the most widespread alteration was found in delays of PSPs in TG neurons, whereas the degree of alterations was the most limited in current transfers and rise times. S*teady state and sinusoidal voltage transfers were also differentially altered*. Alterations were present in 2.5 times higher percentage of intervals in relation to steady-state than sinusoidal voltage transfers in basal dendritic arbors (33.3% vs. 13.3%). Differential alterations in steady-state and sinusoidal voltage transfers may reflect a relative shift or modulation in spread of NMDA- and non–NMDA receptor–mediated PSPs with slower and faster kinetics. Such a differential alteration is supported by direct experimental findings on differential modulation of glutamatergic signaling in neurodegenerative diseases (Di Lazzaro et al., 2003; Revett et al., 2013) and by the fact that memantine, approved to treat moderate and severe forms of AD, modulates disturbed glutamatergic system (Frost et al., 2015).

All the aforementioned features of degrees of alterations showed qualitative and quantitative similarities along absolute and normalized scales, suggesting that our observations on alterations in subthreshold dendritic signaling are independent of the choice of scale in comparison graphs.

### Synaptic Input Pattern Recognition Is Preserved in Tauopathy

In previous phases of our analysis, we explored various tau-related pathological alterations in dendritic propagation of locally generated single PSPs. This was useful because we could systematically study trends in alterations of different aspects of dendritic signaling mediated by the presence of mutant tau protein. However, neurons usually receive not a single, but multiple synaptic potentials, which overlap in time and summate on axon hillock near the cell body. The result of this summation determines firing activity of postsynaptic neurons. Recognition and differentiation between activation patterns of dendritic synapses are a vital process in neuronal information processing, and it is also involved in learning and memory, which are known to be affected in tauopathies. Therefore, we investigated possible alterations in levels of synaptic input pattern recognition capability in TG neurons related to simultaneous activation of multiple synapses. These estimates for levels of synaptic input recognition/discrimination were based on a thorough study by de Sousa et al. (2015). This study, by using an extremely large number of model neurons with different morphologies and with active and passive membranes, concluded that means and variances of electrotonic distances of synapses correlate strongly with level of synaptic input pattern recognition (see section Materials and Methods for a brief summary). Thus, in the next step, we investigated if these simple metrics undergo any changes in neurons of tau-TG mice to assess whether presence of mutant human tau protein leads to pathological alterations in synaptic input pattern recognition.

**FIGURE 12:**
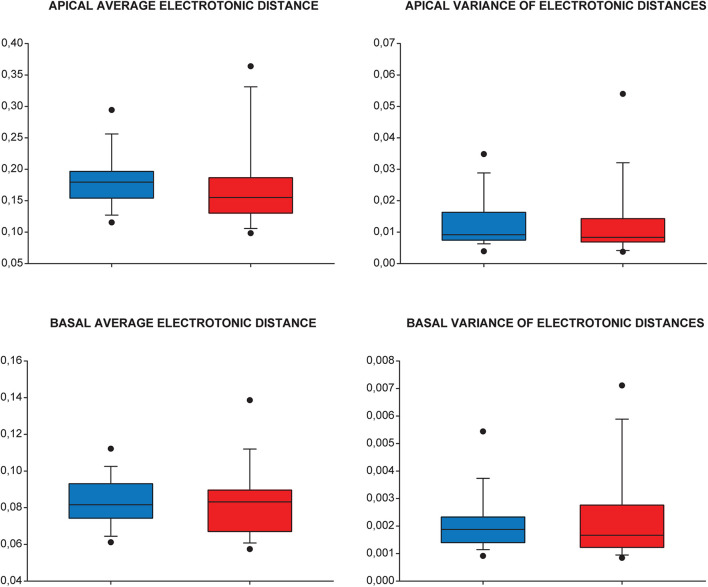
Synaptic input pattern recognition/discrimination is unchanged in advanced tauopathy. Means and variances of electronic distances of modeled dendritic synapses (injection sites) from the cell body were used to quantify and compare levels of synaptic input pattern recognition/discrimination capabilities in WT (blue), TG (red) neurons. Apical (upper panels) and basal (lower panels) dendritic arbors were analyzed separately. Lower and upper boundaries of the boxes indicate the 25th and 75th percentiles; a line within each box marks the median value; whiskers below and above the boxes show the 10th and 90th percentiles; lower and upper closed circles represent the 5th and 95th percentiles. Statistical analysis showed no significant (ns.) alteration in the predictors of synaptic input pattern recognition/discrimination capabilities of TG neurons (Mann–Whitney test, *p* > 0.161, *p* > 0.46, *p* > 0.68, and *p* > 0.93 for apical average and variance and basal average and variance of electrotonic distances, respectively).

Analysis of means and variances of electrotonic distances between modeled synaptic sites and the cell body in WT and TG groups of neurons revealed no statistically significant difference in these critical parameters (Mann–Whitney test, *p* > 0.161 for all cases). This finding suggests that synaptic input pattern recognition/discrimination remains unaltered in advanced tauopathy (Figure 12).

## Discussion

### Unaltered Specific Membrane Resistance and Capacitance in rTg4510 Pyramidal Neurons

When we were building computer models of WT and TG neurons with their reconstructed morphology, we estimated specific membrane resistances (*R*_*m*_) and capacitances (*C*_*m*_) of these model neurons by varying *R*_*m*_ values until electrophysiologically measured neuron resistance is reached and then by varying *C*_*m*_ until experimental membrane time constant was matched in the model neuron. These *R*_*m*_ and *C*_*m*_ values are therefore dependent on both reconstructed morphology and measured physiological properties of respective neurons. The mean specific membrane resistance and capacitance of model TG neurons showed no change relative to control. This finding has multiple important implications.

**FIGURE 13:**
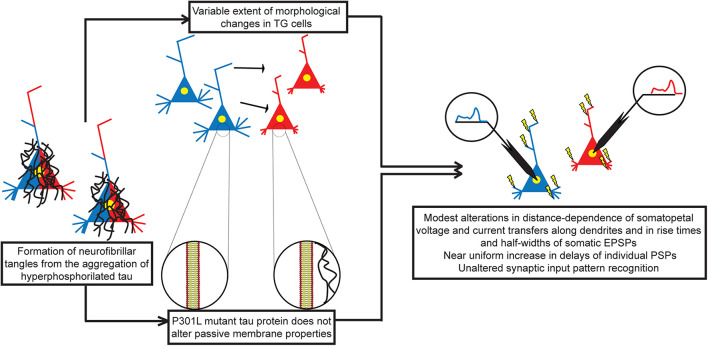
Morphofunctional effects of human P301L mutant tau protein on layer III pyramidal neurons in the rTg4510 mouse model of advanced tauopathy. Mutation in tau protein leads to aggregation and formation of NFTs. Mutant tau protein detaches from microtubules leading to destabilization of the cytoskeleton and atrophy of dendrites. On the other hand, mutant tau protein does not alter passive membrane properties, such as specific membrane resistance and capacitance of neurons. Therefore, detected alterations in subthreshold dendritic signaling of individual PSPs are entirely due to alterations in morphology of tau-mutant neurons. Alterations in voltage and current transfers as well as in rise times and half-widths of somatic EPSPs are limited spatially to PSPs produced by only a fraction of synapses, and these alterations are relatively small in size. Time delays between synaptic activity and somatic voltage response show general significant increase in TG neurons, and sizes of these increases are nearly independent of the dendritic location of synapses. Restricted alterations in transfers/rise times/half-widths and the uniform increase in delays of PSPs may leave synaptic integration unchanged, leading to unaltered input pattern recognition/discrimination in advanced tauopathy. WT neurons in blue, TG neurons in red colors, and neurons with both colors are in the process of getting altered.

First, to our best knowledge, this is the first attempt so far to estimate the effects of a human mutant tau protein on resistance and capacitance of neuronal membranes. Second, predicted unaltered leakage resistance of neuronal membrane is in agreement with the lack of tau-induced membrane permeabilization and channel formations at physiological pH values, reported earlier in a study combining direct biophysical measurements and atomic force microscopy in artificial membranes (Patel et al., 2015). Third, in the absence of tau-dependent alterations of passive membrane properties, and assuming unaltered axial resistance of the cytoplasm, any modification in subthreshold dendritic impulse propagation must be entirely due to tau-induced morphological changes in these TG neurons (Figure 13).

### Unaltered Synaptic Input Pattern Recognition/Discrimination

We predicted unaltered synaptic input pattern recognition/discrimination in layer III pyramidal neurons of the tau-mutant animal. This conservation of synaptic input pattern recognition in TG neurons suggests virtually unaltered summation of multiple subthreshold PSPs. Our prediction on unaltered nature of this summation is also supported by more direct computational evidence, such as spatially limited and minor alterations in voltage/current transfers and in shapes of somatic EPSPs, and the nearly uniform, location-independent increase in delays of individual PSPs shown by our simulations. This way, the size of somatic signals and effective time window for their summation, which are the key factors of synaptic integration, may remain generally unaffected, which may, in turn, lead to unaltered synaptic summation.

In an earlier article, we had shown that synaptic input pattern recognition/discrimination did not get altered in layer III pyramidal neurons of the Tg2576 amyloid mouse model of AD either (Somogyi et al., 2016), where the human amyloid precursor protein with the Swedish mutation was overexpressed, leading to Aβ accumulation. These findings on the absence of alterations in synaptic input pattern recognition under the effect of pathological Aβ and tau, which are two important proteins of many neurodegenerative diseases and both of which are present in AD, may have important message as to the future directions of research toward therapeutics: Once integration of synaptic signals do not get altered by the pathological Aβ and tau proteins, this may indicate that pathological network functions such as seizures and epileptiform activities seen in patients with AD (Amatniek et al., 2006; Lozsadi and Larner, 2006; Scarmeas et al., 2009) and in model animals of AD (Palop et al., 2007; Bezzina et al., 2015) are not primarily due to intrinsic alterations of signaling in neurons of affected networks but linked to network-level pathological changes, for example, loss/rearrangement or modulation of synaptic connections, all of which have been described.

### Voltage and Current Transfers

Our computations revealed spatially limited and relatively small alterations in distance dependence of subthreshold voltage and current transfers of propagating dendritic PSPs (Figures 5–7) in response to tau burden in mutant mice. Another alteration predicted by our simulations is that a relatively bigger fraction of dendritic synapses has increased voltage transfers for their PSPs (Figures 6, 7), increasing the weighted arithmetic mean of steady-state voltage transfer rate of PSPs significantly (i.e., reducing average attenuations of signals). Other area-weighed transfers remained unaltered in tau-mutant neurons. Although the increase in arithmetic mean of steady-state voltage transfers was statistically significant, this alteration represented only a 5% rise relative to WT level. Thus, the biological significance of this increase alone may be small; however, it is in line with previous suggestions on tau-mediated hyperexcitability of neurons in mutant mice (Roberson et al., 2007; Ittner et al., 2010; Devos et al., 2013; Holth et al., 2013) and in humans with tauopathy (Amatniek et al., 2006; Vossel et al., 2013). All this makes the prediction on increased average transfers to be partially responsible for increased excitability more feasible. This possibility is further supported by the consistency in direction of statistically significant alterations in voltage transfers, which were, at all distances, increases rather than decreases in model TG neurons. In addition, P301L mutant tau expression has also been shown to increase glutamate release and reduce glutamate clearance in rTg4510 mice and therefore suggested to underlie neuronal hyperexcitability of neurons (Hunsberger et al., 2015). Our finding on increased efficiency of transfers of voltage transients from dendritic synaptic sites to the soma in the same animal model of tauopathy is another, previously unknown, factor that may contribute to the higher intrinsic excitability of tau-mutant neurons.

### Possible Experimental Paradigms to Confirm the Hypotheses Generated by Our Computer Model

#### Measuring Specific Membrane Capacitance and Resistance

Some of the predictions of our simulation study might be tested experimentally. One of these testable predictions is the unaltered nature of the specific membrane capacitance and resistance in P301L tau-mutant pyramidal neurons. Gentet et al. (2000) developed a direct measurement method to determine specific membrane capacitance in a wide variety of neurons, including neocortical pyramidal cells in rodents. In this method, 300-μm-thick brain slices were used, and following compensation of pipette capacitance in the cell-attached configuration, nucleated patches were pulled from pyramidal neurons. These patches were clamped at −60 mV; −5 mV steps were applied, and capacitive current transients were recorded and averaged and then fitted by a single exponential function with an added constant. Time constant and amplitude were used to estimate capacitance of the membrane patch. Surface area of the nucleated patch was measured from images captured by differential interference contrast infrared microscopy. Finally, specific membrane capacitance could be determined by dividing the capacitance of the membrane patch by its surface area. This method is potentially also suitable for measuring specific membrane resistance of neurons directly. By measuring and comparing membrane properties in WT and TG neurons with the above method, our prediction on unaltered passive membrane properties could be put into experimental test.

Another, potentially suitable method to measure passive membrane properties of neurons was published by Wright et al. (1996). This is a white noise approach based on zero-mean Gaussian white noise current stimuli, which was successfully applied for estimating passive electrical properties of dentate granule cells in whole-cell patch configuration.

#### Probing Transfers and Delays of PSPs

Another prediction by our computational work is that slowdown of somatopetal signal propagation and voltage and current transfers of PSPs in dendrites of tau-mutant neurons remain virtually unaltered. This prediction could be investigated experimentally by simultaneous somatic and dendritic recordings by patch electrodes, while current is injected at a dendritic location. Essentially, the experiments carried out by Williams and Stuart (2002) on neocortical neurons of brain slices could be repeated. They investigated attenuations of artificial EPSPs evoked by current injections through an electrode at various dendritic sites, whereas another somatic electrode recorded the somatic voltage response.

From voltage–time responses recorded this way at dendritic sites and at the soma, while steady-state or sinusoidal current is injected to the dendrite, voltage transfers as a function of distance could be derived. In addition, by using the time course of the injected current, delays between somatic voltage response and current injection could be calculated, just like in our simulations.

In these experiments, double dendritic electrodes with minimal distance separation should be applied to inject current independently and measure dendritic depolarization avoiding filtering and adding an offset during current injections (Williams and Stuart, 2002). In such experimental attempts several other special cautions must also be taken as summarized in a detailed review on dendritic patch clamp recordings by Davie et al. (2006).

Recording membrane potential dynamics by microelectrodes provide an excellent temporal resolution, but the number of recording sites (spatial resolution) is limited, and dendrites with small calibers remain largely inaccessible. However, new imaging techniques may provide a less invasive, suitable combination of spatial and temporal resolution to analyze membrane potential changes with high throughput at subcellular level in the foreseeable future. Currently, calcium imaging suffers from the problem of slow kinetic of calcium transients relative to the underlying membrane potential dynamics. This problem is further complicated by the difficulty of relating calcium transients to voltage changes as reviewed by Kulkarni and Miller (2017). Direct voltage imaging techniques have recently shown several new inventions to improve spatiotemporal resolution and brightness of the signal, including the use of novel fluorescent proteins (Jin et al., 2012; St-Pierre et al., 2014), opsins (Hochbaum et al., 2014; Gong et al., 2015), second-harmonic generation (Reeve et al., 2013), and nanomaterials (Marshall and Schnitzer, 2013; Park et al., 2013). Some of these techniques may soon provide a way to measure voltage transfers and time delays of propagating subthreshold dendritic signals in finer, more distal dendrites of neurons.

#### Testing Synaptic Integration

Comparing synaptic input pattern recognition/discrimination in WT and TG neurons by direct laboratory experiments is difficult. However, this ability of neurons is strongly interrelated with more testable features of synaptic integration, such as coincidence detection and effect of input timing of subthreshold synaptic inputs on action potential output. Timing and precision of spike initiation in layer V pyramidal cells of the rat somatosensory cortex have been investigated in brain slices (Berger and Luscher, 2003). In this experiment, a pair of extracellular bipolar electrodes was used to induce two independent or partially overlapping sets of excitatory and inhibitory PSPs in the same pyramidal neuron. Delay between the two sets of inputs was varied. During induction of synaptic inputs, recordings were made from the soma to record the output of the postsynaptic neuron. Simultaneous recordings from the soma and dendrites allowed estimation of the approximate location of the activated synapses. This way, the authors could study integration and coincidence detection of two sets of synaptic inputs to pyramidal neurons. In this analysis, the output was measured by mean spiking probability in the postsynaptic neuron.

In another analysis, coincidence detection in pyramidal neurons was investigated by injecting computer-generated current trains simulating synaptic currents arriving from two input sources (Grande et al., 2004). Delay was systematically varied between the two simulated synaptic inputs, while whole-cell recordings were made from the soma to assess firing rate as a function of phase delay between the two inputs.

Similar comparative experiments on cortical slices from WT and TG mice could shed some light on the presence or absence of tau-induced alterations in synaptic integration.

### Possible Consequences of Mutant-Tau-Driven Alterations at Neuronal Circuitry Level

Our simulations predicted subthreshold voltage and current transfers with only spatially localized, small-sized alterations and increased intraneuronal signal delays with very little location dependence in tau-mutant neurons. Statistically significant alterations in rise times and half-width of somatic EPSPs were also rarely detected when the dendritic site of PSP generation was varied. These predictions combined with unaltered predictors of synaptic input pattern recognition/discrimination forecast unaltered synaptic integration. However, in reality, neurons are building blocks of neural networks with rich and diverse connection patterns. If the spatiotemporal pattern of synaptic input changes, it may lead to altered action potential output, even if integrative properties of the postsynaptic neuron are unchanged. Spatiotemporal activation pattern of synaptic input may change because of loss/alterations of synaptic connections and also because of the different temporal pattern of synaptic activation of existing connections. Loss and alteration of synaptic contacts have been described; alteration in temporal pattern is likely in mutant-tau-protein–affected cortical networks. On the other hand, intraneuronal signaling delays can add up in a series of coupled neurons causing a substantially delayed firing in upstream neurons of networks. In case of feedback or converging/diverging connections, such accumulation of delays in action potential generation may be different in converging pathways and may therefore alter temporal pattern of synaptic inputs to downstream neurons. Such a mechanism may lead to devastating degree of alteration in the activity of the whole neural network, even if intraneuronal determinants of synaptic integration and spatial pattern of synaptic inputs remain unaltered.

This mutant human tau-protein–induced toxic mechanism, which leads to delayed and/or completely degraded network function, is speculative but it is based on predictions of our simulations and results in pathological network activity known to exist both in animals and humans with tauopathies.

### Relation to Previous Modeling Studies

One major prediction of our simulations is the slowdown of dendritic PSP propagation in TG neurons. This slowdown may have multiple reasons. Considering anomalous axial diffusion, a theoretical study also predicted a slowdown of PSP propagation along dendrites in neurons with reduced spine densities, which is characteristic in many neurodegenerative disorders, including Frontotemporal Dementia (FTD) (Henry et al., 2008). This study used fractional cable model to account for anomalous axial diffusion of ions and molecules in dendrites. Such diffusion is involved in electrochemical signaling of neurons; thus, alterations in this diffusion may have an impact on electrotonic and firing properties of neurons and also on delays of PSPs propagating along dendrites (Langlands et al., 2009). Degree of this anomaly in axial diffusion is associated with the density/number/shape of spines, as spines trap and release molecules as revealed by experimental studies that visualized diffusion in spiny dendrites of hippocampal CA1 pyramidal neurons and Purkinje cells (Santamaria et al., 2006, 2011). Beyond spines, alterations in speed of signaling may also be caused by changes in other, non–spine-related morphological features of dendrites and also by changes in intracellular and extracellular diffusion due to deposition of amyloid plaques, a hallmark of AD (Mueggler et al., 2004; Banks and Fradin, 2005). Amyloid plaques do not appear in rTg4510 mice, but dendritic atrophy is well-documented at the age of 9 months (Rocher et al., 2010; Crimins et al., 2012).

Our neuron models of rTg4510 neurons accounted for the loss of spines and other morphological alterations of dendrites but did not account for the anomalous diffusion in spiny dendrites. Spine loss alone, without considering other dendritic alterations and anomalous diffusion, is expected to speed up dendritic signaling toward the soma (Agmon-Snir and Segev, 1993). Reason for this is the decrease in dendritic surface area due to loss of spines that affects effective specific membrane resistances and capacitances in our cable models. The fact that our cable models of TG neurons, in the absence of anomalous diffusion, showed a slowdown of dendritic signal propagation suggests that tau-driven, but non–spine-related morphological alterations (e.g., in diameters or topology of dendrites, etc.) play a role in slowdown of signaling in rTg4510 pyramidal neurons. As the net effect of morphological alterations is an increase rather than a decrease of delays in our TG neuron models, and passive membrane properties are not affected by mutant tau protein, this indicates that the increase in delays may be caused by non–spine-related morphological alterations, whose effect overweighed the effect of decreased spine density in TG neurons. Indeed, e.g., changes in diameters of dendrites can alter propagation the speed of signals (Agmon-Snir and Segev, 1993), and alterations in dendritic diameters of rTg4510 mice have been described (Rocher et al., 2010; Crimins et al., 2011).

Regarding descriptors of dendritic signaling, other than delays, the effects of morphology on amplitudes and rise times of somatic EPSPs have also been studied in computational models of healthy hippocampal CA3 pyramidal neurons in rats (Henze et al., 1996). In another study, by using passive, anatomically realistic compartmental models, Kabaso et al. (2009) computed voltage attenuations in neocortical layer II/III pyramidal neurons of young and old monkeys. These authors found that somatopetal and somatofugal voltage attenuations generally were reduced (voltage transfers were increased) because of morphological changes in older neurons. Major changes in voltage attenuations occurred in apical rather than in basal dendritic arbors. Increased voltage transfers in older neurons were concluded to potentially lead to their increased excitability, and these single-cell level alterations may contribute to age-related cognitive decline. In this context, it is interesting to note that we also found a statistically significant increase in area-weighed mean steady-state voltage transfers in pyramidal neurons of tau-mutant mice, but unlike in aging, we found that in tauopathy voltage transfers were altered more in basal than in apical arbors.

Finally, the importance of general dendritic morphology in determination of functional properties of pyramidal neurons is further emphasized by another model study that found dendritic diameters to affect firing rate of neurons more than active membrane properties in certain scenarios (Weaver and Wearne, 2008).

### Significance of Subthreshold Membrane Models

Distribution of voltage-dependent conductances in plasma membrane of layer III pyramidal neurons of the frontal lobe has not been studied in WT and rTg4510 mice in a comparative manner. Therefore, we had to restrict our analysis to subthreshold signaling. However, dendrites of neocortical pyramidal neurons are known to be endowed with voltage-dependent conductances, although most of these data are from layer V pyramidal neurons because they are easier to access experimentally because of their thicker dendrites. Layer III pyramidal neurons have been reported to have actively back-propagating action potentials and bear voltage-dependent sodium channels (Waters et al., 2003; Waters and Helmchen, 2006), whose activity is accompanied by influx of calcium ions (Svoboda et al., 1999). Hyperpolarization-activated, cyclic nucleotide-gated channels are likely to have important contribution to signaling properties in pyramidal neurons of rTg4510 mice, as their activation is related to the significantly increased sag potentials and excitability in TG neurons (Crimins et al., 2012). This is likely because somatic voltage changes, in response to current injections at dendritic sites, are affected by *I*_*h*_ channels in layer V pyramidal neurons (Williams and Stuart, 2000). While this previous experimental work investigated the effect of *I*_*h*_ channels on somatic response in healthy neurons, our simulations predicted alterations in dendritic signaling due to tau-driven dendritic atrophy but in the lack of I_*h*_ channels. Absence of voltage-dependent channels in our computer models is a clear limitation. Future computational studies on tau-mutant neurons should also consider the effect of *I*_*h*_ and other voltage-dependent channels on dendritic signaling, once reliable data on these channels from TG neurons become available.

On the other hand, the possible importance of subthreshold depolarizations and their summation in layer III pyramidal neurons is emphasized by the sparse firing activity combined with large fluctuations in membrane potentials observed below the firing threshold in layer III pyramidal neurons (Sakata and Harris, 2009; Gentet et al., 2010; Crochet et al., 2011).

Finally, although we studied propagation of PSPs with passive membrane restriction, some of our results may be relevant to certain aspects of the dendritic signaling, when voltage-gated ion channels do open (active membrane). Our prediction on unaltered synaptic input pattern recognition/discrimination in TG neurons remains valid in case of active membrane as well. This is because the two metrics (mean and variance of electrotonic distances of synapses) we used to predict possible changes in synaptic input pattern recognition/discrimination had been shown to be valid predictors of synaptic input pattern recognition/discrimination in neurons with active membrane properties too (de Sousa et al., 2015). Further, regarding attenuation of passively back-propagating action potentials, somatopetal current transfers we calculated in passive membrane model are identical to values of somatofugal voltage transfers (Zador et al., 1995); hence, somatopetal current transfers are related to attenuation of back-propagating action potentials too.

### Comparison of the Effects of Aβ and Mutant Tau on Neuronal Membrane and Dendritic Signaling

Somogyi et al. (2016) presented a similar analysis on how Aβ affects neuronal membranes and dendritic signaling in Tg2576 amyloid mouse model of AD in the absence of mutant tau. This earlier and our recent analyses suggest the two key proteins to affect layer III neocortical pyramidal neurons remarkably differently, leaving synaptic input pattern recognition unaltered in both cases. It was found that Aβ decreases membrane resistance and increases membrane capacitance (Somogyi et al., 2016), whereas in our current article, we report mutant tau protein not to change membrane resistance and capacitance in TG neurons. Aβ was shown to affect the morphology and membrane properties of pyramidal neurons in a way that morphological and membrane alterations compensate each other’s pathological effects and subthreshold dendritic signaling, and the input pattern recognition/discrimination remains virtually unaltered (Somogyi et al., 2016). Such a compensation of dendritic atrophy by parallel alterations in membrane properties is not possible in rTg4510 mice because of the unaltered nature of passive membrane properties. This means that, in the tau-TG animal, conservation of synaptic input pattern recognition at control level must be due to compensatory effects among the various alterations in morphology (e.g., lengths, diameters, topology, spine densities) of dendrites. Details of this mechanism remain to be elucidated in the future.

Although it is useful to separate the effects of these two key proteins of neurodegenerative diseases to understand their distinct impacts on dendritic signaling, on the other hand, their synergistic effects (Rapoport et al., 2002; Blurton-Jones and Laferia, 2006; Atsmon-Raz and Miller, 2015) on membranes and morphology cannot be excluded when they act simultaneously as in patients with AD. Probably the most relevant of these synergistic effects to our study is that presence of Aβ peptide leads to increased phosphorylation and aggregation of tau protein and therefore facilitates tau pathology (Gotz et al., 2001; Lewis et al., 2001; Blurton-Jones and Laferia, 2006; He et al., 2018). Hence, one possible logical step in this series of computer simulation studies would be analysis of alterations in dendritic signaling properties under simultaneous effects of Aβ and mutant tau proteins in neurons of double-TG animals.

## Data Availability Statement

Program codes are available upon reasonable request to corresponding author or via ModelDb database.

## Ethics Statement

Ethical review and approval was not required for the animal study because this study did not directly involve any human or animal subjects since our theoretical study was based on cellular morphological and electrophysiological data that are from the NeuroMorpho.org database and from published manuscript.

## Author Contributions

AS wrote simulation codes, performed computer simulations, analyzed the data, created the figures, and contributed to revising the manuscript. EW contributed to statistics, figures and to analysis of results, designed and organized the study, discussed and interpreted results, and wrote the manuscript. Both authors contributed to the article and approved the submitted version.

## Conflict of Interest

The authors declare that the research was conducted in the absence of any commercial or financial relationships that could be construed as a potential conflict of interest.

## Publisher’s Note

All claims expressed in this article are solely those of the authors and do not necessarily represent those of their affiliated organizations, or those of the publisher, the editors and the reviewers. Any product that may be evaluated in this article, or claim that may be made by its manufacturer, is not guaranteed or endorsed by the publisher.

## Footnotes

1
Neuromorpho.org
